# Safety Profile of Rapamycin Perfluorocarbon Nanoparticles for Preventing Cisplatin-Induced Kidney Injury

**DOI:** 10.3390/nano12030336

**Published:** 2022-01-21

**Authors:** Qingyu Zhou, Justin Doherty, Antonina Akk, Luke E. Springer, Ping Fan, Ivan Spasojevic, Ganesh V. Halade, Huanghe Yang, Christine T. N. Pham, Samuel A. Wickline, Hua Pan

**Affiliations:** 1 Department of Pharmaceutical Sciences, Taneja College of Pharmacy, University of South Florida, Tampa, FL 33612, USA; 2 USF Health Heart Institute, University of South Florida, Tampa, FL 33602, USA; justindohert@usf.edu (J.D.); ghalade@usf.edu (G.V.H.); wickline@usf.edu (S.A.W.); 3 Department of Medicine, Washington University School of Medicine, St. Louis, MO 63110, USA; antoninaakk@wustl.edu (A.A.); lspringer@wustl.edu (L.E.S.); cpham@wustl.edu (C.T.N.P.); 4 School of Medicine, Duke University, Durham, NC 27708, USA; ping.fan@duke.edu (P.F.); ivan.spasojevic@duke.edu (I.S.); huanghe.yang@duke.edu (H.Y.); 5 John Cochran Veterans Affairs Medical Center, St. Louis, MO 63106, USA; 6 Altamira Therapeutics Inc., Dover, DE 19901, USA

**Keywords:** rapamycin, perfluorocarbon nanoparticle, autophagy, inflammation, pharmacokinetics, biodistribution, kidney function, cardiac function, spleen, immune responses

## Abstract

Cancer treatment-induced toxicities may restrict maximal effective dosing for treatment and cancer survivors’ quality of life. It is critical to develop novel strategies that mitigate treatment-induced toxicity without affecting the efficacy of anti-cancer therapies. Rapamycin is a macrolide with anti-cancer properties, but its clinical application has been hindered, partly by unfavorable bioavailability, pharmacokinetics, and side effects. As a result, significant efforts have been undertaken to develop a variety of nano-delivery systems for the effective and safe administration of rapamycin. While the efficacy of nanostructures carrying rapamycin has been studied intensively, the pharmacokinetics, biodistribution, and safety remain to be investigated. In this study, we demonstrate the potential for rapamycin perfluorocarbon (PFC) nanoparticles to mitigate cisplatin-induced acute kidney injury with a single preventative dose. Evaluations of pharmacokinetics and biodistribution suggest that the PFC nanoparticle delivery system improves rapamycin pharmacokinetics. The safety of rapamycin PFC nanoparticles was shown both in vitro and in vivo. After a single dose, no disturbance was observed in blood tests or cardiac functional evaluations. Repeated dosing of rapamycin PFC nanoparticles did not affect overall spleen T cell proliferation and responses to stimulation, although it significantly decreased the number of Foxp3^+^CD4^+^ T cells and NK1.1^+^ cells were observed.

## 1. Introduction

Cancer treatment-induced toxicity occurs not only in patients during their anti-cancer therapies but also in cancer survivors many years after the treatment is completed. Based on NCI statistics, there are more than 16.9 million cancer survivors in the United States [1] and over 21.7 million expected survivors by 2029 [2]. Cancer treatment successes have been hampered by disproportionate incidences of other diseases caused by anti-cancer treatments, leading to excessive and unanticipated morbidity and mortality in this population [3,4,5,6,7]. For cancer patients receiving anti-cancer therapies, treatment-induced toxicities may necessitate the discontinuation of effective therapy, typically resulting in undertreatment [8,9]. Cisplatin, discovered over 50 years ago [10], remains one of the most potent anti-cancer chemotherapy drugs and is used as first-line chemotherapy in ~10–20% of all cancer patients [11]. However, the full anti-cancer potential of cisplatin remains underutilized due to treatment-induced toxicities, particularly nephrotoxicity [12,13,14]. A single cisplatin dose in the range of 50–100 mg/m^2^ results in nephrotoxicity in ~30% of patients [15], and 50–70% of patients receiving a five-day course of cisplatin at 15–20 mg/m^2^/day develop nephrotoxicity [16]. Therefore, nephrotoxicity is a major limiting factor for cancer patients receiving effective cisplatin treatment. Moreover, patients recovering from acute kidney injury from supportive measures are at a 25% increased risk of developing chronic kidney disease and a 50% increased risk of ten-year mortality [17,18,19]. The lasting adverse effects of cisplatin-induced nephrotoxicity were highlighted by Skinner R et al. in a 10-year follow-up study on childhood cancer patient survival [20]. Therefore, the development of new strategies to mitigate cisplatin-induced acute kidney injury could significantly improve the outcomes of cancer treatment and survivors’ quality of life.

Rapamycin is a macrolide that was originally isolated from *Streptomyces hygroscopicus* in a soil sample from Easter Island, or Rapa Nui [21,22,23]. It is well documented that rapamycin is a potent mTOR-dependent inducer of autophagy [24,25,26]. Rapamycin-induced mTOR inhibition not only enhances autophagy but also inhibits inflammation by blocking mTOR downstream NF-κB signaling [27]. It has been reported that the inhibition of NF-κB signaling pathway activation alleviates cisplatin-induced kidney injury [28]. Furthermore, accumulating evidence suggests that inducing autophagy could be beneficial in mitigating cisplatin-induced kidney injury [29]. Therefore, it is worth the effort to investigate the therapeutic potential of rapamycin in protecting renal injury from cisplatin treatment. Currently, rapamycin is FDA approved as an oral immunosuppressant to prevent transplant rejection by blocking interleukin-2 signaling in B- and T-cells [30]. Although rapamycin offers significant potential for treating a wide range of pathological and physiological conditions, from vascular restenosis, inflammation, and cancer to aging, its clinical applications have been hindered partially by unfavorable bioavailability and pharmacokinetics. To improve its therapeutic utility, significant efforts in the nanomedicine field have been devoted to developing a variety of nano-delivery systems, including micelles [31,32,33], liposomes [34,35], polymers [36], and PFC nanoparticles [37,38]. However, full analyses of the pharmacokinetics, biodistribution, and safety of nanomedicine preparations remain to be performed. Herein, we build on our previous studies on the effectiveness of rapamycin PFC nanoparticle treatment of vascular restenosis [37] and aging [38] to assess its potential for preventing cisplatin-induced kidney injury. Furthermore, we performed comprehensive evaluations on the pharmacokinetics, biodistribution, and toxicity studies of these rapamycin PFC nanoparticles for potential clinical translation.

## 2. Materials and Methods

### 2.1. Rapamycin Perfluorocarbon (PFC) Formulation and Characterization

Rapamycin PFC nanoparticles were formulated by using methods described previously [39], with modifications. Briefly, a lipid/rapamycin mixture of 98.6 mol% egg lecithin, 1 mol% dipalmitoyl-phosphatidylethanolamine (Avanti Polar Lipids, Piscataway, NJ, USA), and 0.4 mol% rapamycin (Cat# J62473, Alfa Aesar from FisherSci, Tampa, FL, USA) was dissolved in the mixture of methanol and chloroform (1:3, *v/v*). The solvents were removed under reduced pressure for generating a lipid film with rapamycin, which was dried in a vacuum oven overnight. Next, the lipid film containing rapamycin (2.0%, *w/v*), PFC (Gateway Specialty Chemicals, St. Peters, MO, USA) (20%, *w/v*), and MilliQ water were sonified and emulsified at 20,000 psi for six passes in an ice bath (LV-1 Microfluidics emulsifier; Microfluidics, Newton, MA, USA). The size distributions of the rapamycin PFC nanoparticles were evaluated by dynamic light scattering (Brookhaven Instruments Corp., Holtsville, NY, USA). The surface charges of the nanoparticles, indicated by zeta-potential values, were determined with a PALS Zeta Potential Analyzer (Brookhaven Instruments Corp.). Data were collected in the mode of phase-analysis light-scattering (PALS) after solution equilibrated at 25 °C. The loading of the rapamycin on PFC nanoparticles was determined by using LC/MS/MS assay on rapamycin PFC nanoparticles after 72 h dialysis [37]. LC/MS/MS assay is described in detail below. 

### 2.2. Western Blot

NRK-52E cells, rat kidney proximal tubule cells (CRL-1571, ATCC, Manassas, VA, USA), were seeded in 6 well plates at a density of 100,000 cells per well. Twenty-four hours post-seeding, cells were treated with or without rapamycin PFC nanoparticles at 0.1 µg/mL. Twenty-four hours later, for the cisplatin groups, cells were treated with cisplatin at a dose of 25 µM. Twenty-four hours post-cisplatin treatment, all the cells were collected for protein extractions. RIPA buffer (R0278-500ML; Sigma-Aldrich, St. Louis, MO, USA) with one tablet of protease inhibitors (4906837001; Sigma-Aldrich) per 10 mL RIPA buffer and PMSF (8553; Cell Signaling Technology, Danvers, MA, USA) at a final concentration of 1 mM were prepared for extracting proteins. Briefly, cells were disrupted in the aforementioned protein extraction buffer and protein lysates collected by centrifugation for 10 min at 12,000× *g* at 4 °C. Protein concentration was assessed with BCA protein assay (23225; Thermo Fisher Scientific, Waltham, MA, USA). Under reducing conditions, equivalent amounts of total protein were fractionated by using SDS-gel electrophoresis. Membranes were probed with rabbit anti-p62, anti-p65, anti-Actin, or anti-Bax (1:1000 dilution, ab109012, Abcam; 8242S, cell signaling Technology; ab8227, Abcam; and ab182734, Abcam, respectively) overnight at 4 °C. Membranes were then washed and incubated with secondary antibody anti-rabbit HRP (1:10,000 dilution, sc-2313; Santa Cruz Biotechnology, Dallas, TX, USA) at room temperature for one hour. Bands were visualized by using Pierce ECL Western blotting substrate (32106; Thermo Fisher Scientific) with ChemiDoc MP (Bio-Rad Laboratories, Hercules, CA, USA). 

### 2.3. Cell Viability Evaluation

2F-2B, mouse endothelial cells, or NRK-52E cells were seeded at 100,000 cells/well in 6 well plates. Twenty-four hours post-seeding, cells were treated with or without free rapamycin or rapamycin PFC nanoparticles at indicated concentrations of rapamycin. Twenty-four hours post-treatment, cells were collected and evaluated in the fluorescence mode of a LUNA-FL dual fluorescence cell counter (Logos Biosystems, Annandale, VA, USA). 

### 2.4. Animal Procedures

#### 2.4.1. Evaluation of Therapeutics in Mitigating Cisplatin Induce Acute Kidney Injury

Male C57BL/6 mice (10–12 weeks old) received saline, free rapamycin, or rapamycin PFC nanoparticles at 0.1 mg rapamycin/kg. Twenty-four hours later, mice received cisplatin i.p. injections at 10 mg/kg. Mice without any treatment served as control. Blood was collected at sequential time points after cisplatin treatment for selected analyses. 

#### 2.4.2. Single Intravenous Administration of Unformulated Free Rapamycin and Rapamycin-Loaded Nanoparticles for Pharmacokinetic Analysis

Rapamycin used for intravenous (IV) administration was dissolved in Tween 80/polyethylene glycol (50:50, *v/v*) at a concentration of 1 mg/mL, and then diluted with saline to obtain the solution with a finial concentration of 0.1 mg/mL prior to use. Rapamycin nanoparticles contained 0.1 mg/mL of rapamycin. Male C57BL/6 mice (10–12 weeks old) were randomly divided into two groups: unformulated free rapamycin (0.1 mg/kg), and rapamycin nanoparticle (0.1 mg/kg) groups. Prior to the IV administration of unformulated free rapamycin and rapamycin nanoparticles, the left carotid artery of each animal was catheterized for IV dosing and blood sampling. Each animal was then given a single IV bolus injection of 30 μL of rapamycin solution or rapamycin nanoparticle suspension through the left carotid artery and the catheter was flushed with 100 μL of saline. Whole blood samples were collected at 0 min (pre-dose) and 5, 20, and 40 min, and 1, 2, 4, 6, 8 and 20 h post-dose. An aliquot of 20 μL of whole blood sample was immediately mixed with 0.4 μL of 0.5 M potassium EDTA and stored at −80 °C before drug analysis. Following the blood sampling at 20 h post-dose, all animals were euthanized under isoflurane anesthesia and perfused to purge the vasculature of residual blood. Heart, lung, liver, kidney, spleen, stomach, small intestine, brain and bladder were collected at autopsy and stored at −80 °C before LC-MS/MS analysis of rapamycin concentrations in tissues.

#### 2.4.3. Effects of Free Rapamycin or Rapamycin Nanoparticles in Immune Responses

Mice received either free rapamycin or rapamycin nanoparticle at 0.1 mg of rapamycin/kg once a week for four weeks. To evaluate the effects of the treatment on T cell responses, spleens were collected 24 h after the last nanoparticle injection and stimulated ex vivo. To test for anti-nanoparticle antibody formation, blood was collected from the inferior vena cava 30 min after the last nanoparticle injection and the levels of anti-rapamycin nanoparticle antibody were assayed. 

#### 2.4.4. Blood Test

Twenty-four hours post-treatment, blood was collected via cardiac puncture for complete blood count and blood chemistry tests at Department of Comparative Medicine Research Animal Diagnostic Laboratory at Washington University School Medicine. For complete blood count, 50 µL whole blood was collected into Ethylenediaminetetraacetic acid (EDTA) test tube. Blood chemistry tests were performed on serum (n = 5 per group).

#### 2.4.5. Blood Urea Nitrogen (BUN) Test

BUN test was performed by using Urea Nitrogen (BUN) Colorimetric Detection Kit (K024-H5, Arbor Assays, Ann Arbor, MI, USA) and following the user manual. The animal procedures and protocols were approved by the Institutional Animal Care and Use Committee at the University of South Florida. 

### 2.5. LC/MS/MS Assay of Rapamycin in Nanoparticles, Blood, and Tissue

#### 2.5.1. Sample Processing

Samples were thawed and homogenized with two parts water (w/vol) and two 2.5 mm zirconia/silica beads (Biospec Products Inc., Bartlesville, OK, USA) in a blunted 0.5 mL polypropylene (PP) conical vial by vigorous agitation in Fast-Prep apparatus (Thermo-Savant. Thermo Fisher Scientific) at speed 4 for 40 s at room temperature. A 100 µL aliquot of the obtained tissue homogenate, 10 µL of 200 ng/mL desmethoxyrapamycin, DMR (Internal standard. Supelco, Inc. Bellefonte, PA, USA), and 300 µL chloroform were added into 1.5-mL PP conical vial and vigorously agitated in Fast-Prep apparatus (speed 4, 40 s). After discarding the (upper) aqueous phase, 200 µL of the organic phase was transferred to 12 × 75 mm glass tube and evaporated to dryness by gentle stream of nitrogen, the residue reconstituted with 50 µL precipitate mix (70% methanol, 30% 0.3 M ZnSO_4_ in water) and transferred into LC/MS/MS injection vial. In case of blood, 10 µL of blood, 10 µL of 200 ng/mL DMR, and 40 µL of precipitate mix (70% methanol, 30% 0.3 M ZnSO_4_ in water) were added into 0.2-mL PCR tube and vigorously agitated in Fast-Prep apparatus (speed 4, 40 s). After centrifugation at 13,600× *g* for 5 min at room temperature, 40 µL of supernatant was transferred into injection vial for analysis.

#### 2.5.2. Liquid Chromatography-Tandem Mass Spectrometry (LC-MS/MS)

The LC-MS/MS analysis of rapamycin was performed on a Shimadzu 20A series LC system interfaced with Applied Biosystems/SCIEX API 4000 QTrap MS/MS spectrometer (AB Sciex LLC, Framingham, MA, USA). Analyst software (version 1.6.1) was used for mass parameters tuning, data acquisition, and quantification. LC conditions were: (1) column: Phenomenex Kinetex (C18, 3 × 4 mm, #AJ0-4287. Phenomenex. Torrance, CA, USA); (2) mobile phase A: 10 mM ammonium acetate, 0.1% acetic acid, 3% methanol, in DI water; (3) mobile phase B: 10 mM ammonium acetate, 0.1% acetic acid, 3% DI water, in methanol; (4) flow rate: 0.5 mL/min; (5) elution gradient: 0–1 min, 50% B; 1.0–1.5 min, 50–100% B; 1.5–2.0 min, 100% B, 2.0–2.2 min, 100–50% B; (6) run time: 3.5 min. A diverter valve was used to send flow to MS/MS only between 0.6 and 2 min. Autosampler at 4 °C; injection volume was 20 µL. The compounds were individually infused as 100 ng/mL solutions in 50%A/50%B at 10 µL/min flow rate and ionization/ion path parameters were optimized. Parent/daughter quantifier (qualifier) ions utilized were: rapamycin (931.5/864.5(882.4)) and DMR (901.5/852.5(834.5)).

#### 2.5.3. Calibration and Quantification

Calibration samples (n = 6) were prepared by adding pure standard (rapamycin, Supelco) of the measured compound to tissue homogenate in the appropriate range. The following are ranges used (the lower value representing also the LLOQ at 80% accuracy limit, all other calibrator levels at 85% accuracy limit): 2.4–1000 ng/mL (blood) and 0.7–90 ng/mL (tissue). Signal area integration, calibration, and quantification were performed within Analyst v 1.6.1 software. The response of the peak area standard/internal standard to nominal concentration was linear with r = 0.999 or better.

### 2.6. Pharmacokinetic Analysis

A two-compartment open model with first-order elimination from the central compartment ($C=Ae^{-\alpha t}+Be^{-\beta t}$) was used to characterize rapamycin disposition in blood, where *α* and *β* represent the first-order fast and slow disposition rate constants, respectively, and *A* and *B* are the corresponding zero-time intercepts. The half-lives for the fast (*t*_1/2,*α*_) and slow (*t*_1/2,*β*_) disposition phase were calculated as $t_{1/2,\alpha }=\frac{0.693}{\alpha }$ and $t_{1/2,\beta }=\frac{0.693}{\beta }$, respectively. Volume of the central compartment (*V_C_*) is calculated as $V_{C}=\frac{D}{A+B}$, where D is the dose administered. Volume of distribution at steady state (*V_ss_*) is calculated as $V_{ss}=\frac{D\left[AUMC\right]}{{\left[AUC\right]}^{2}}$, in which total area under the blood concentration-time curve (*AUC*) is calculated as $AUC=\frac{A}{\alpha }+\frac{B}{\beta }$. Total area under the first moment curve (*AUMC*) is calculated as $AUMC=\frac{A}{\alpha^{2}}+\frac{B}{\beta^{2}}$. Volume of distribution of the slow disposition phase (*V_β_*) is calculated as $V_{\beta }=\frac{D\times \beta }{AUC}$. Total clearance (*CL*) is calculated as $CL=\frac{D}{AUC}$. 

### 2.7. Transthoracic Echocardiography and Data Analyses

Mice were anesthetized using isoflurane (2–3%) in a closed chamber before being placed on a temperature-maintained platform in the supine position. Echocardiographic images of male C57BL/6 mice (10–12 weeks old) were acquired using a VisualSonics Vevo 3100-LAXR-X under steady-state isoflurane anesthesia (1.0–1.5%). Ultrasound images were acquired with a high-resolution transducer (MX400, axial resolution: 50 μm). Mouse heart rates (~450 beats/min) were monitored continuously. After 3–5 min of physiological stabilization of temperature and heart rate, image acquisition proceeded in long and short axis B- and M-mode. Three consecutive image analyses were performed by the sonographer blinded to groups, as previously reported [40]. 

### 2.8. Splenocytes Isolation and Stimulation

Spleens were collected aseptically with scissors, minced in cold PBS and passed through nylon mesh filters to obtain an homogenous cell suspension. The erythrocytes were lysed by the addition of Tris-NH4Cl, washed twice with cold PBS, and counted by trypan blue. Viability was >95%.

Ninety-six-well U-bottom microculture plates (Corning Inc., Corning, NY, USA) were coated with anti-CD3 (5 µg/mL, 145-2C11; eBioscience, San Diego, CA, USA) in PBS overnight. Plates were washed twice with PBS prior adding cells. Briefly, splenocytes were added into 96 well U-bottom microculture plates at 1 × 10^5^ cells/well in a complete medium RPMI 1640 supplemented with 10% (*v/v*) FBS (Gibco), 1% (*v/v*) NEAA, 1% (*v/v*) sodium pyruvate, 2 mM L-glutamine, 10 mM Hepes, 50 µM 2-mercaptoethanol, 100 µg/mL streptomycin, 100 units/mL penicillin (all from Sigma-Aldrich).

Cells were activated in plate-bound anti-CD3 (5 µg/mL, 145-2C11; eBioscience). All tests were carried out in triplicate. The plates were incubated at 37 °C in a humidified atmosphere with 5% CO_2_ for 72 h.

### 2.9. Cytokine Assays and Cell Proliferation

Following 72 h of incubation, culture supernatants were harvested, and cytokine concentrations determined by BD™ Cytometric Bead Array (CBA), Mouse Inflammation Kit, cat# 552,364, according to manufacturer’s protocol. Cells were harvested and acquired at BD FACSCalibur™ for 45 s to define fluorescence intensities over time. Cell proliferation was analyzed using BD CellQuest™ Pro software. All assays were performed in triplicates.

### 2.10. Flow Cytometry

Antibodies against the following molecules coupled to the indicated fluorochromes were used from BD Pharmingen or eBioscience: PE anti-NK1.1 (PK136; eBioscience), FITC anti-CD4, PerCP anti-CD4 (L3T4; BD Pharmingen, San Diego, CA, USA), FITC anti-CD19 (1D3; BD Pharmingen), PerCP anti-CD8, APC anti-CD8 (53-6.7; BD Pharmingen, San Diego, CA, USA), PerCP CD4/80 (BM8, eBioscience).

In general, 10^6^ cells were blocked with the anti-FcR mAb 2.4G2, stained with the indicated antibodies for 20 min at 4 °C and then washed and resuspended for FACS analysis. For Foxp3 expression analysis, the Foxp3 Staining Kit (cat# 88-8118-40, eBioscience) was used according to manufacturer’s instructions. Flow cytometry was performed on the BD FACSCalibur™. Data analysis was performed using BD CellQuest™ Pro software.

### 2.11. Anti-Nanoparticle Antibody Formation

Plates were coated with rapamycin or rapamycin nanoparticles overnight. The next day plates were washed three times with 0.05% tween-20 in PBS, blocked with 1% BSA/PBS for 1 h, and washed again three times. Serum samples were diluted 1:100 in 1% BSA/PBS; 100 μL was added to each well and incubated for 2 h at room temperature. After washing three times, 100 μL of either IgG or IgM HRP conjugated antibody (goat anti-mouse IgG/IgM-HRP, Cat #’s: 1030-05, 1020-05. Southern Biotechnology Associates. Birmingham, AL, USA) was added to each well at a 1:3000 dilution and incubated for 2 h at room temperature. The plates were washed and developed according to the protocol of the substrate manufacturer (Substrate Reagent Pack, Cat No. DY999, R&D Systems, Minneapolis, MN, USA) and read at 450 nm absorbance using a Spectra Max Plus.

### 2.12. Statistics

Unless described in other sections, results were expressed as mean ± standard error of mean (SEM). Statistical analyses of pharmacokinetic data were performed using Graph Pad Prism 8.0 (Graph Pad, San Diego, CA, USA). Comparison of means between two independent groups was made using the two-sample *t* test. One-way ANOVA with Bonferroni post-hoc testing was used. Statistical significance of differences was attributed at *p* < 0.05.

## 3. Results

### 3.1. Physical Characterization of Rapamycine Perfluorocarbon (PFC) Nanoparticles

**Figure 1:**
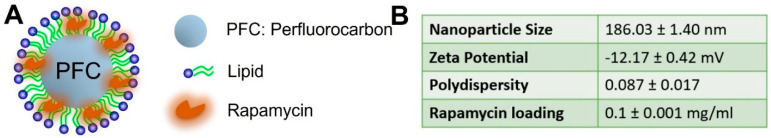
Physical characterization of rapamycin PFC nanoparticles. (**A**) A schematic of rapamycin PFC nanoparticle, which is composed of a hydrophobic perfluorocarbon (PFC) core surrounded by a lipid monolayer carrying rapamycin. (**B**) Nanoparticle size, zeta potential, polydispersity index, drug loading features of rapamycin PFC nanoparticles. n = 3 Data are presented as mean ± SEM.

Perfluorocarbon (PFC) nanoparticles are dual-function theranostic vehicles that are potentially useful for both noninvasive imaging and drug delivery [41]. PFC nanoparticles consist of a hydrophobic perfluorocarbon core surrounded by a lipid monolayer, as shown in Figure 1A. The lipid monolayer can be functionalized by incorporating targeting ligands, imaging agents, and/or therapeutic payloads. To add rapamycin as therapeutic payloads, we formulated rapamycin into the lipid monolayer, taking advantage of the hydrophobic nature of rapamycin. In a previous study, we demonstrated that the lipid monolayer of the PFC nanoparticles served as a preferred carrier for hydrophobic rapamycin with stable drug retention of 97%, when tested against an infinite sink [37]. As shown in Figure 1B, rapamycin PFC nanoparticles exhibited a diameter of 186.03 ± 1.40 nm, zeta potential of −12.17 ± 0.42 mV, and polydispersity index of 0.087 ± 0.017. The rapamycin loading was 0.1 ± 0.001 mg/mL. 

### 3.2. Rapamycin PFC Nanoparticles Provide Therapeutic Benefits in Cisplatin-Induced Acute Kidney Injury

**Figure 2:**
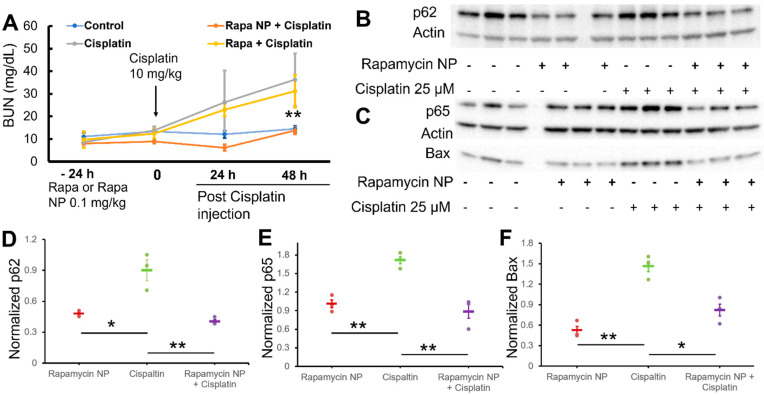
Pretreatment of rapamycin PFC nanoparticles mitigates cisplatin induced acute kidney injury. (**A**) BUN from control mice, cisplatin-exposed (cisplatin), pretreatment of free rapamycin followed with cisplatin exposure (rapa + cisplatin), pretreatment of rapamycin PFC nanoparticle followed with cisplatin exposure (rapa NP + cisplatin) groups (n = 5 per group). (**B**) Western blot results of p62, an indicator of autophagy, from NRK-52E cell lysates in the indicated treatment groups. (**C**) Western blot results of p65 and Bax, indicators of inflammatory response and apoptosis, from NRK-52E cell lysates in the indicated treatment groups. (**D**) Semi-quantification of p62 western blot results, normalized to the control group (n = 3). (**E**) Semi-quantification of p65 western blot results, normalized to the control group (n = 3). (**F**) Semi-quantification of Bax western blot results, normalized to the control group (n = 3). Significance: * *p* < 0.05; ** *p* < 0.01. Data are presented as mean ± SEM.

To evaluate the therapeutic potential of rapamycin for mitigating cisplatin-induced acute kidney injury, we pretreated mice with either free rapamycin or rapamycin PFC nanoparticles 24 h prior to 10 mg/kg cisplatin injections. Mice without any treatment or with only cisplatin injection served as controls. Blood was collected serially from each mouse for blood urea nitrogen (BUN) analysis to assess kidney function. As demonstrated in Figure 2A, for the control group, the serial BUN data were: 11.04 ± 1.69 mg/dL, 13.29 ± 0.56 mg/dL, 12.10 ± 1.58 mg/dL, and 14.48 ± 1.37 mg/dL. For the group receiving rapamycin PFC nanoparticles pretreatment, the BUN data at the same time points were: 7.99 ± 1.79 mg/dL, 8.92 ± 0.98 mg/dL, 6.13 ± 1.42 mg/dL, and 13.76 ± 1.61 mg/dL. For the group receiving rapamycin pretreatment, the BUN data were: 9.69 ± 3.69 mg/dL, 12.32 ± 2.16 mg/dL, 22.91 ± 2.87 mg/dL, and 31.12 ± 7.24 mg/dL. For the group receiving only cisplatin injection, the BUN data were: 8.58 ± 1.36 mg/dL, 13.76 ± 1.73 mg/dL, 26.22 ± 14.05 mg/dL, and 36.28 ± 11.63 mg/dL. The results demonstrate that cisplatin injection at 10 mg/kg induced substantial kidney damage that was mitigated by rapamycin PFC nanoparticle treatment, compared to free rapamycin treatment (*p* < 0.001). 

To examine the mechanisms potentially responsible for this beneficial effect, we employed renal proximal tubule cells (NRK-52E) in vitro that were subjected to cisplatin injury and rapamycin treatments. Exposure to rapamycin PFC nanoparticles 24 h before cisplatin incubation (25 µM) elicited: (1) significantly enhanced autophagy (Figure 2B), where the p62 level was significantly reduced in the groups with the rapamycin nanoparticle treatment; (2) reduced cisplatin-induced inflammation through NF-κB signaling pathway activation, where p65 upregulation was only observed in the group receiving cisplatin exposure; and (3) reduced apoptosis (Figure 2C), where rapamycin pre-treatment significantly inhibited cisplatin-induced Bax expression. The results of the semi-quantification by Western blot normalized to controls (i.e., cells without any treatment) are presented in Figure 2D–F. As shown in Figure 2D, rapamycin PFC nanoparticles enhanced autophagy irrespective of cisplatin treatment according to reduced p62 levels: 0.90 ± 0.10 for cisplatin without rapamycin treatments (control); 0.41 ± 0.02 or 0.48 ± 0.02, for rapamycin NP or rapamycin NP + cisplatin, respectively, *p* = NS. Significant enhancements in autophagy were noted for both rapamycin NP and rapamycin NP + cisplatin groups: *p* = 0.015 and *p* = 0.009, respectively (Figure 2D).

Pretreatment with rapamycin PFC nanoparticles inhibited cisplatin-induced NF-κB activation according to reduced p65 protein levels (0.888 ± 0.110 vs. 1.714 ± 0.057, rapamycin NP + cisplatin vs. cisplatin, *p* = 0.007) (Figure 2E). For the cells receiving only rapamycin PFC nanoparticles, the normalized p65 was 1.012 ± 0.060, which was significantly lower than in the cisplatin group (1.714 ± 0.057) (*p* = 0.003) (Figure 2E). Bax, a pro-apoptotic protein, was also evaluated among all groups. The results suggested that rapamycin PFC nanoparticle pre-treatment, significantly reduced Bax levels in the cells exposed to cisplatin (0.822 ± 0.086 vs. 1.460 ± 0.077, rapamycin NP + cisplatin vs. cisplatin, *p* = 0.013) (Figure 2F). For the cells receiving only rapamycin PFC nanoparticles, normalized Bax was 0.525 ± 0.055, which was significantly lower than in the cisplatin group (*p* = 0.002), illustrating that rapamycin PFC nanoparticle treatment reduces pro-apoptotic protein levels 24 h after treatment.

### 3.3. Neither Free Rapamycin Nor Rapamycin PFC Nanoparticles Affect Cell Viability

**Figure 3:**
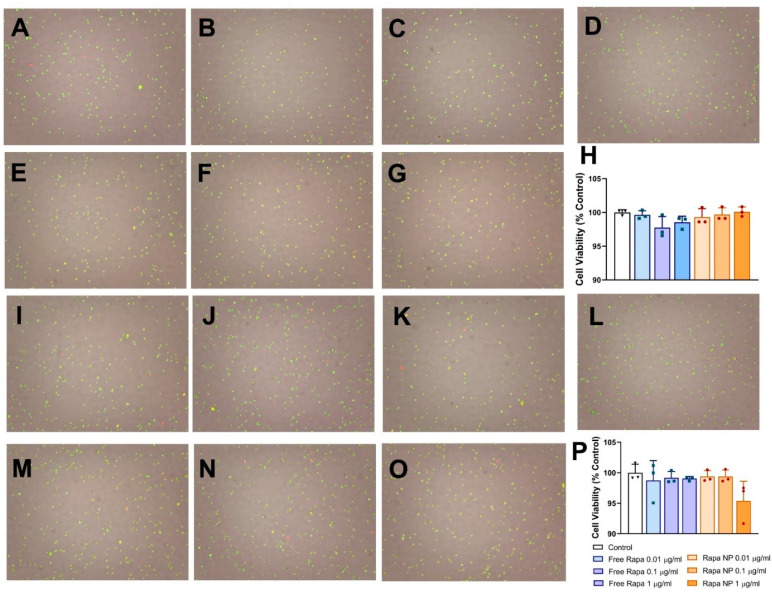
Effect of free rapamycin or rapamycin PFC nanoparticles on endothelial and proximal tubule cells viability. (**A**–**C**) Representative cell viability photographs of endothelial cells (2F-2B) treated with rapamycin PFC nanoparticles at rapamycin concentrations of 0.01, 0.1, and 1 µg/mL, respectively. (**D**) Representative photograph of endothelial cells (2F-2B) without treatment serving as control. (**E**–**G**) Representative photographs of endothelial cells (2F-2B) treated with free rapamycin at concentrations of 0.01, 0.1, and 1 µg/mL, respectively. (**H**) Quantification of 2F-2B cell viability. (**I**–**K**) Representative photographs of proximal tubule cells (NRK-52E) treated with rapamycin PFC nanoparticles at rapamycin concentrations of 0.01, 0.1, and 1 µg/mL, respectively. (**L**) Representative photograph of proximal tubule cells (NRK-52E) without treatment serving as control. (**M**–**O**) Representative photographs of proximal tubule cells (NRK-52E) treated with free rapamycin at concentrations of 0.01, 0.1, and 1 µg/mL, respectively. (**P**) Quantification of NRK-52E cell viability (n = 3 per group). Data are presented as mean ± SEM. (Control: ▽; Free rapamycin: □; Rapa NP: ○.).

To assess the safety of the treatment in vitro, we treated endothelial cells (2F-2B) or proximal tubule cells (NRK-52E) with either free rapamycin or rapamycin PFC nanoparticles and evaluated the cell viability 24 h post-treatment. As illustrated in Figure 3A–C,H, 24 h after the rapamycin PFC nanoparticle treatment of 2F-2B cells at rapamycin concentrations of 0.01, 0.1, or 1 µg/mL, the cell viabilities were 99.32 ± 1.23%, 99.69 ± 1.00%, or 100.10 ± 0.71%, respectively. For the free rapamycin treatment, the cell viabilities were 99.63 ± 0.64%, 97.76 ± 1.64%, or 98.54 ± 0.91%, respectively (Figure 3D–H). The results suggest that free rapamycin and rapamycin PFC nanoparticles do not compromise endothelial cell viability at these rapamycin dosages. As presented in Figure 3I–K,P, 24 h after the rapamycin nanoparticle treatment of NRK-52E cells at rapamycin concentrations of 0.01, 0.1, or 1 µg/mL, the cell viabilities were 99.38 ± 0.95%, 99.38 ± 1.05%, or 95.38 ± 3.24%, respectively. For the free rapamycin treatment, the cell viabilities were 98.73 ± 3.26%, 99.18 ± 1.04%, or 99.04 ± 0.36%, respectively (Figure 3M–P). These results suggested that at the tested dosages, neither rapamycin nor rapamycin PFC nanoparticles impair proximal tubule cell viability. 

### 3.4. Differential Kinetics and Distribution of Free Rapamycin and Rapamycin PFC Nanoparticles

**Figure 4:**
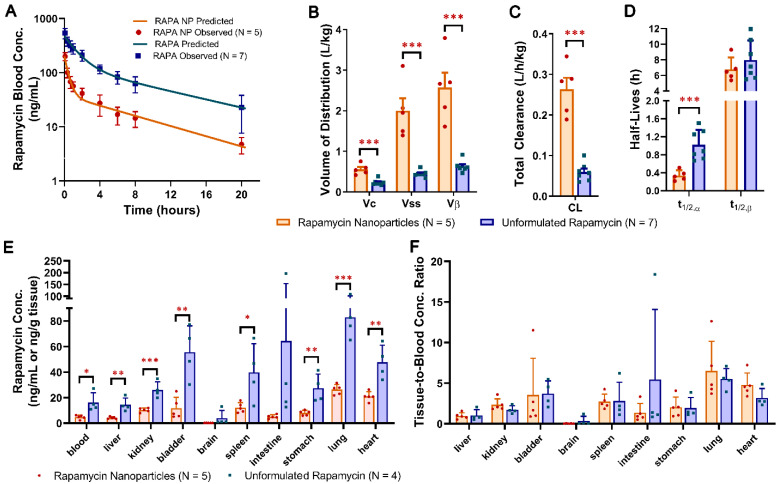
Systemic disposition kinetics and tissue distribution of rapamycin following intravenous (IV) administration of 0.1 mg/kg of unformulated rapamycin or rapamycin nanoparticles in C57BL/6 mice. (**A**) A bi-exponential decline in rapamycin blood concentrations in the rapamycin blood concentration-time profile after the IV dosing of either unformulated rapamycin (n = 7) or rapamycin nanoparticles (n = 5) to C57BL/6 mice. (**B**) Rapamycin nanoparticles (n = 5) exhibited significantly higher volume of the central compartment (*V_C_*), volume of distribution at steady state (*V_ss_*), and volume of distribution of the slow disposition phase (*V_β_*) than the unformulated rapamycin (n = 7) (*p* < 0.001 for all). (**C**) Rapamycin nanoparticles (n = 5) exhibited significantly higher total clearance (*CL*) than the unformulated rapamycin (n = 7) (*p* < 0.001). (**D**) Rapamycin nanoparticles (n = 5) exhibited significantly shorter half-life for the fast disposition phase (*t*_1/2,*α*_) than the unformulated rapamycin (n = 7) (*p* < 0.001). No significant difference in half-life for the slow disposition phase (*t*_1/2,*β*_) was observed between unformulated rapamycin and rapamycin nanoparticle groups. (**E**) At 20 h after the IV dosing of 0.1 mg/kg of unformulated rapamycin or rapamycin nanoparticles, the mean rapamycin concentrations in blood (*p* < 0.05), liver (*p* < 0.01), kidney (*p* < 0.001), bladder (*p* < 0.01), spleen (*p* < 0.05), stomach (*p* < 0.01), lung (*p* < 0.001), and heart (*p* < 0.01) were significantly higher in the unformulated rapamycin group (n = 4) compared with the rapamycin nanoparticle group (n = 5). (**F**) No significant difference in the mean rapamycin tissue-to-blood concentration ratios was found between unformulated rapamycin and rapamycin nanoparticle groups. Data are expressed as mean ± standard deviation (SD). SD is denoted by the error bars. Comparison of mean values between the unformulated rapamycin and rapamycin nanoparticle groups was made using the two-sample *t* test. * *p* < 0.05, ** *p* < 0.01, and *** *p* < 0.001 compared with the unformulated rapamycin group.

The systemic disposition kinetics of rapamycin in C57BL/6 mice following a single IV bolus injection of 0.1 mg/kg of unformulated free rapamycin or rapamycin nanoparticles was best described by the two-compartmental open model with first-order elimination from the central compartment (Figure 4A). The results of the PK analysis showed that the mean *V_C_*, *V_ss_*, and *V_β_* values of the rapamycin nanoparticle group were 1.4 fold (0.557 ± 0.129 L/kg vs. 0.237 ± 0.071 L/kg), 3.5-fold (2.00 ± 0.678 L/kg vs. 0.448 ± 0.083 L/kg) and 3.1 fold (2.57 ± 0.817 vs. 0.633 ± 0.137 L/kg) higher than those of the unformulated free rapamycin group (*p* < 0.01 for all), suggesting that the use of rapamycin-loaded nanoparticles greatly improves the extent of rapamycin distribution in tissues (Figure 4B; Supplemental Table S1). Similarly, rapamycin’s total clearance of the nanoparticle group was increased by 3.1 fold (0.263 ± 0.063 L/h/kg vs. 0.060 ± 0.022 L/h/kg) compared with that of the unformulated free rapamycin group (*p* < 0.001. Figure 4C). This fold change value was roughly comparable to the difference observed in *V_ss_* and *V_β_* between the two study groups, indicating that rapamycin nanoparticles increased the relative tissue distribution and elimination of rapamycin to the same extent. As a result, the difference in the half-life of the slow disposition phase (*t*_1/2,*β*_) was not significant between the rapamycin nanoparticle and the unformulated free rapamycin groups (6.78 ± 1.51 h vs. 7.97 ± 2.51 h. *p* > 0.05. Figure 4D; Supplemental Table S1). Nonetheless, the half-life of the fast disposition phase (*t*_1/2,*α*_) of the nanoparticle group was twofold shorter than that of the unformulated free rapamycin group (0.34 ± 0.12 h vs. 1.02 ± 0.33 h. *p* < 0.001. Figure 4D; Supplemental Table S1). 

The rapamycin concentrations in blood and different tissues at 20 h after the IV dosing of unformulated free rapamycin or rapamycin nanoparticles and the tissue-to-blood concentration ratios are shown in Figure 4E and Figure 4F, respectively. Comparisons of rapamycin blood and tissue concentrations between the two study groups showed that rapamycin concentrations in the blood (*p* < 0.05), liver (*p* < 0.01), kidney (*p* < 0.001), bladder (*p* < 0.01), spleen (*p* < 0.01), stomach (*p* < 0.01), lung (*p* < 0.001), and heart (*p* < 0.01) were significantly lower in the rapamycin nanoparticle group compared with the unformulated free rapamycin group (Figure 4E; Supplemental Table S2). However, there was no significant difference in tissue-to-blood concentration ratio in any tissue type between the two study groups (*p* = NS; Figure 4F), suggesting that the observed difference in rapamycin concentrations in tissues is attributable to the dissimilar systemic exposure exhibited by unformulated free rapamycin and rapamycin nanoparticles. 

Although the slow decline in rapamycin blood concentrations during the terminal elimination phase (i.e., the *β* phase) enables direct comparisons between tissue concentrations, analysis of the ratio of tissue to blood rapamycin concentrations provides more accurate assessment of drug distribution because any differences between blood concentrations are accounted for in the ratio. In this regard, the relatively low observed rapamycin tissue concentrations in the rapamycin nanoparticle group was likely attributable to the relatively low rapamycin blood concentrations at 20 h post-dosing (16.2 ± 7.6 ng/mL (nanoparticle) vs. 4.7 ±1.6 ng/mL (unformulated), *p* < 0.05). Nevertheless, the rapamycin tissue distribution pattern appeared to be different between the two study groups. In the unformulated free rapamycin group, rapamycin was preferentially distributed in descending order in the lung, intestine, bladder, heart, stomach, kidneys, liver, and brain, whereas in the rapamycin nanoparticle group, rapamycin was preferentially distributed in descending order in the lung, heart, bladder, spleen, kidneys, stomach, intestine, liver, and brain. It is postulated that the physicochemical properties of rapamycin change when incorporated into the non-targeted perfluorocarbon (PFC), which results in an effective change in the permeability of rapamycin, which subsequently affects the drug penetration into different tissues and tissue compartments to various degrees. 

### 3.5. Safety Profile after One Dose of Administration

To define the acute in vivo safety profile, male C57BL/6 mice received either free rapamycin or rapamycin PFC nanoparticles at a dose of 0.1 mg rapamycin/kg, and blood was collected 24 h post-injection for blood cell counts, serum enzyme, and electrolyte evaluations. Hank’s Balanced Salt Solution (HBSS) injection served as the control. Reported in Table 1 are the blood cell/platelet counts, liver/kidney function, and electrolyte profiles of free rapamycin or rapamycin PFC nanoparticle treatment at the given dosage. 

**Figure 5:**
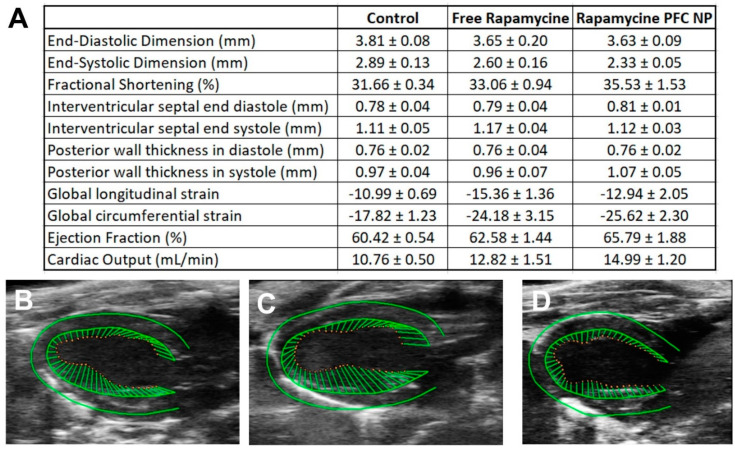
Effects of free rapamycin or rapamycin PFC nanoparticle treatment on cardiac function 24 h after rapamycin dosing. (**A**) Parameters of cardiac function among control mice, the mice receiving free rapamycin, and the mice receiving rapamycin PFC nanoparticle treatment (n = 5 per group). Data are presented as mean ± SEM. (**B**–**D**) Representative long-axis echocardiographic images from control, free rapamycin-treated, and rapamycin PFC nanoparticle-treated mice with strain analyses superimposed.

To assess the effect of the treatment on left ventricular function, we performed transthoracic echocardiography on male C57BL/6 mice 24 h after either free rapamycin or rapamycin PFC nanoparticles at 0.1 mg rapamycin/kg. Male C57BL/6 mice without any treatment served as the control. As indicated in Figure 5A, neither free rapamycin nor rapamycin PFC nanoparticle treatments affected left ventricular function. Figure 5B–D are representative long-axis views of transthoracic echocardiography from control, free rapamycin, or rapamycin PFC nanoparticle-treated mice, respectively.

### 3.6. Effects of Chronic Treatment with Rapamycin PFC Nanoparticles on Immune Responses

**Figure 6:**
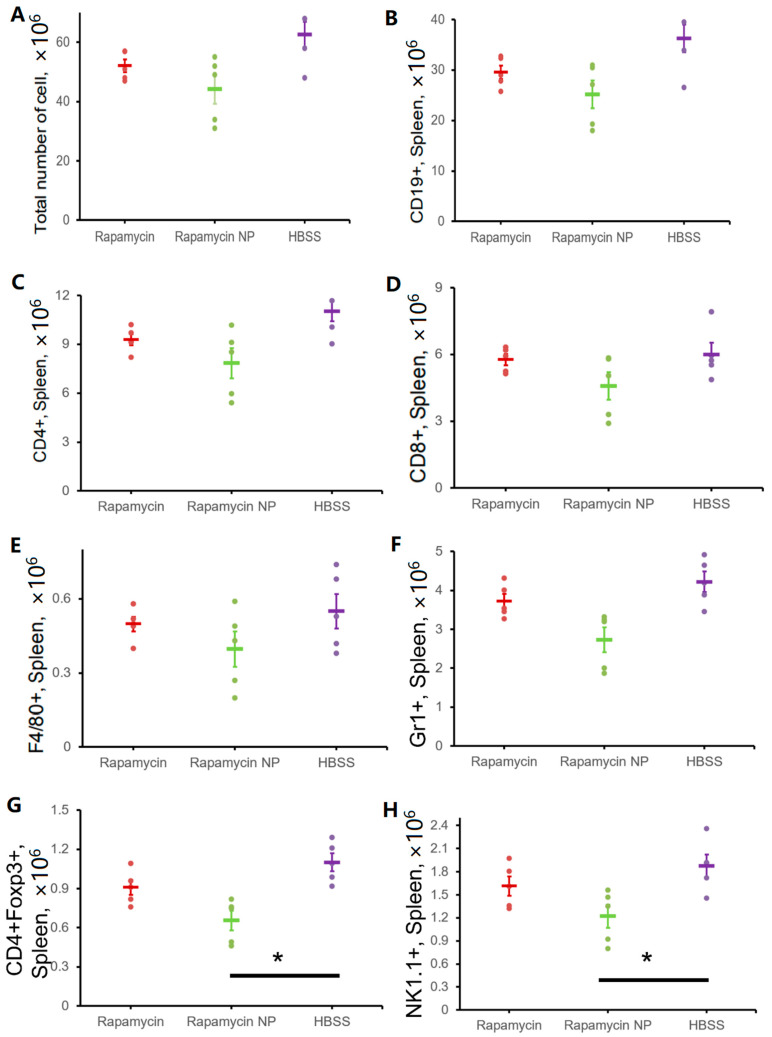
Effects of free rapamycin or rapamycin PFC nanoparticles treatments on spleen cells. Shown are: number of total splenocytes (**A**), C19^+^ cells (**B**), CD4^+^ cells (**C**), CD8^+^ cells (**D**), F4/80^+^ cells (**E**), Gr1^+^ (**F**), CD4^+^Foxp3^+^ cells (**G**), and NK1.1^+^ cells (**H**) (n = 5 per group). Data are presented as mean ± SEM. * *p* < 0.05.

To examine the immunosuppressive properties of rapamycin after chronic administration, we performed repeated dosing, once a week, for four weeks, and evaluated the effects on immune responses for total immune cell populations in the spleen as well as in subpopulation. The results indicate that treatment did not alter the total number of spleen cells (52.00 ± 2.14 × 10^6^ vs. 44.20 ± 4.89 × 10^6^ vs. 62.60 ± 4.26 × 10^6^, rapamycin vs. rapamycin PFC nanoparticles vs. Control (HBSS)) (Figure 6A). Similarly unaltered were subpopulations of CD19+ cells (29.55 ± 1.33 × 10^6^ vs. 25.14 ± 2.76 × 10^6^ vs. 36.26 ± 2.75 × 10^6^, rapamycin vs. rapamycin PFC nanoparticles vs. HBSS) (Figure 6B), CD4^+^ cells (9.28 ± 0.33 × 10^6^ vs. 7.84 ± 0.92 × 10^6^ vs. 11.05 ± 0.64 × 10^6^, rapamycin vs. rapamycin PFC nanoparticles vs. HBSS) (Figure 6C), CD8+ cells (5.76 ± 0.24 × 10^6^ vs. 4.58 ± 0.62 × 10^6^ vs. 6.00 ± 0.51 × 10^6^, rapamycin vs. rapamycin PFC nanoparticles vs. HBSS) (Figure 6D), F4/80^+^ cells (0.50 ± 0.03 × 10^6^ vs. 0.40 ± 0.07 × 10^6^ vs. 0.55 ± 0.07 × 10^6^, rapamycin vs. rapamycin PFC nanoparticles vs. HBSS) (Figure 6E), and Gr1^+^ cells (3.72 ± 0.19 × 10^6^ vs. 2.73 ± 0.32 × 10^6^ vs. 4.22 ± 0.26 × 10^6^, rapamycin vs. rapamycin PFC nanoparticles vs. HBSS) (Figure 6F). However, rapamycin PFC nanoparticle treatment significantly reduced the number of CD4^+^Foxp3^+^ cells (Figure 6G) (0.66 ± 0.08 × 10^6^ vs. 1.10 ± 0.07 × 10^6^, *p* = 0.002) and NK1.1^+^ cells (Figure 6H) (1.22 ± 0.15 × 10^6^ vs. 1.87 ± 0.15 × 10^6^, *p* = 0.01) compared to the control group. 

**Figure 7:**
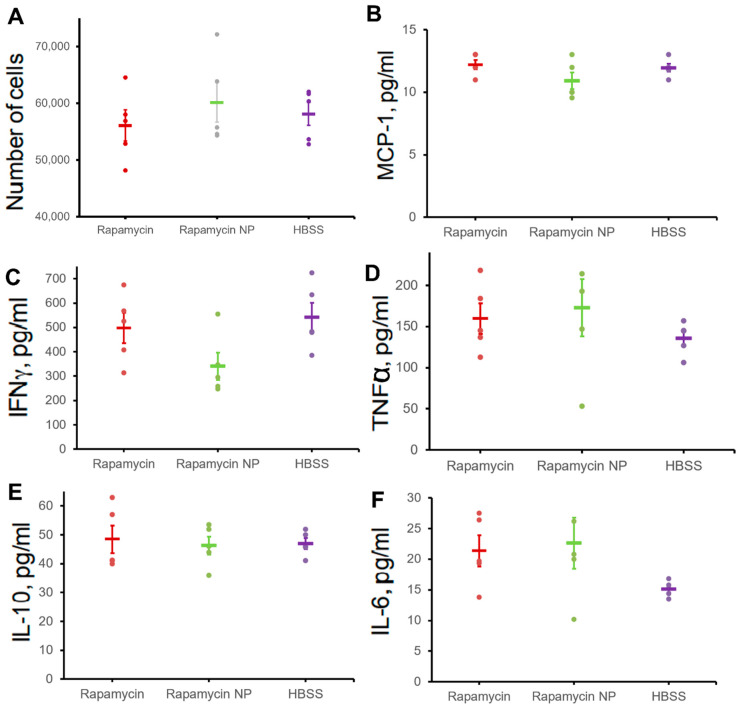
Effects of free rapamycin or rapamycin PFC nanoparticles on proliferation and cytokine production in spleen cells after stimulation of CD3. (**A**) CD4^+^ splenocyte proliferation. Splenic CD4^+^ T cells were stimulated with anti-CD3 monoclonal antibody and following cytokine levels were measured 72 h post stimulation, MCP-1 (**B**), IFNγ (**C**), TNFα (**D**), IL-10 (**E**), IL-6 (**F**) (n = 5 per group). Data are presented as mean ± SEM.

T cells isolated from spleens were further evaluated for their proliferation and cytokine production upon CD3 stimulation. As illustrated in Figure 7, comparing to control (HBSS), the treatment of rapamycin and rapamycin nanoparticles did not affect cell proliferation potential (56105 ± 2737 vs. 60,154 ± 3480 vs. 58,106 ± 2015, rapamycin vs. rapamycin PFC nanoparticles vs. HBSS) (Figure 7A). The expression of cytokines was also not affected: MCP-1 (12.20 ± 0.37 pg/mL vs. 10.91 ± 0.67 pg/mL vs. 11.96 ± 0.32 pg/mL, rapamycin vs. rapamycin PFC nanoparticles vs. HBSS) (Figure 7B), IFN-γ (497.80 ± 62.89 pg/mL vs. 340.20 ± 56.42 pg/mL vs. 541.20 ± 60.65 pg/mL, rapamycin vs. rapamycin PFC nanoparticles vs. HBSS) (Figure 7C), TNF-α (159.40 ± 18.58 pg/mL vs. 172.88 ± 34.73 pg/mL vs. 135.92 ± 8.88 pg/mL, rapamycin vs. rapamycin PFC nanoparticles vs. HBSS) (Figure 7D), IL-10 (48.45 ± 4.81 pg/mL vs. 46.29 ± 3.12 pg/mL vs. 46.94 ± 1.91 pg/mL, rapamycin vs. rapamycin PFC nanoparticles vs. HBSS) (Figure 7E), and IL-6(21.34 ± 2.52 pg/mL vs. 22.60 ± 4.19 pg/mL vs. 15.16 ± 0.57 pg/mL, rapamycin vs. rapamycin PFC nanoparticles vs. HBSS) (Figure 7F). 

**Figure 8:**
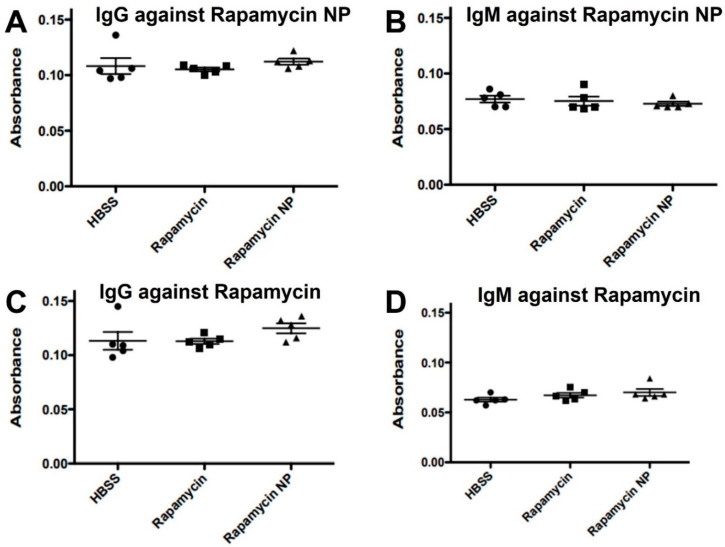
Effect of free rapamycin or rapamycin PFC nanoparticle treatment on humoral responses. IgG (**A**) and IgM (**B**) specific for rapamycin PFC nanoparticles following repeated administration (once a week for four weeks) treatment were not detected in mouse serum. IgG (**C**) and IgM (**D**) specific for free rapamycin following repeated administration (once a week for four weeks) were not detected in mouse serum. Treatments did not elicit adaptive immunoresponse (n = 5 per group) Data are presented as mean ± SEM.

We also investigated the potential of rapamycin FPC nanoparticles to elicit antibody production with repeated administration. As indicated in Figure 8, repeated treatment with rapamycin PFC nanoparticles did not elicit antibody responses against rapamycin or rapamycin PFC nanoparticles. The IgG levels against rapamycin PFC nanoparticles in the mice receiving HBSS (control), rapamycin, or rapamycin PFC nanoparticles were 0.108 ± 0.007, 0.105 ± 0.002, and 0.112 ± 0.003, respectively (Figure 8A). The IgM levels against rapamycin PFC nanoparticles in mice receiving HBSS, rapamycin, or rapamycin PFC nanoparticles were 0.077 ± 0.003, 0.075 ± 0.004, and 0.073 ± 0.002, respectively (Figure 8B). The IgG levels against rapamycin the mice receiving HBSS, rapamycin, or rapamycin PFC nanoparticles were 0.113 ± 0.008, 0.113 ± 0.003, and 0.125 ± 0.005, respectively (Figure 8C). The IgM levels against rapamycin in the mice receiving HBSS, rapamycin, or rapamycin PFC nanoparticles were 0.063 ± 0.002, 0.067 ± 0.002, and 0.070 ± 0.004, respectively (Figure 8D).

## 4. Discussion

In this study, we formulated rapamycin PFC nanoparticles by loading hydrophobic rapamycin onto the lipid surfactant monolayer surrounding PFC-core of the nanoparticles and performed a comprehensive physical characterization and safety analyses. The sizes of the therapeutic PFC nanoparticles (186.03 ± 1.40 nm) were in line with those we reported previously for similar agents, indicating the good reproducibility of the formulation process [38]. The polydispersity measure serves as an index of size distribution, ranging from 0 to 1, where 0 indicates a perfectly monodisperse distribution, and 1 suggests a greatly polydisperse sample with heterogenous particle sizes. In general, the polydispersity index of 0.2 or less is accepted in the field for the size distribution of nanomaterials [42]. Here, our rapamycin PFC nanoparticles featured a polydispersity index of 0.087 ± 0.017, which confirms an acceptably uniform preparation. Furthermore, the zeta potential of −12.17 ± 0.42 mV suggests that the surface charge is sufficiently strong to maintain stability. 

For efficacy, we evaluated the effects of rapamycin formulations on kidney function after cisplatin exposure using either free rapamycin or rapamycin PFC nanoparticles as prophylactic agents. As illustrated in Figure 2A, at 48 h post-cisplatin exposure, the BUN of the mice exposed only to cisplatin was 36.28 ± 11.63 mg/dL, which is greater than 33 mg/dL and confirmed acute kidney injury. Our results suggest that free rapamycin pretreatment provides a minor protective benefit. By contrast, rapamycin PFC nanoparticle pretreatment provided a more potent preventative therapy in cisplatin-induced acute kidney injury compared with free rapamycin pretreatment. 

As to the mechanism responsible for this effect, we employed NRK-52E kidney cell lines to investigate selected molecular responses after cisplatin exposure. The results suggest that rapamycin PFC nanoparticles might simultaneously enhance autophagy and inhibited inflammation in a coordinated manner via the suppression of NF-κB signaling pathway. We have previously shown, in mdx mice with muscular dystrophy (dystrophin-deficient), that rapamycin-loaded PFC nanoparticles can be trapped in damaged and inflamed muscle and heart tissues after IV administration, where they serve as a depot for the slow and sustained release of the drug in high local quantities, which can improve muscular strength in part by limiting autophagy [38]. Moreover, we also reported that PFC nanoparticles functionalized with thrombin inhibitors can be trapped in damaged renal parenchyma after ischemia-reperfusion injury to serve as a sustained beneficial anti-inflammatory treatment [43]. It is intriguing that rapamycin PFC nanoparticles provided better renal protection than free rapamycin, despite both partitioning to similar levels in the kidney. Compared to free rapamycin, rapamycin loaded into PFC nanoparticles could exert different local effects on tissues due to its slow release from the lipid surfactant membrane of the PFC nanoparticles. Consequently, we propose that pretreatment with rapamycin PFC nanoparticles might promote renal proximal tubule cell survival by preventing apoptosis through more effective depot delivery of rapamycin than can be achieved with systemic delivery of the free drug. However, we also note the possibility that other indirect mechanisms could be contributory, such as rapamycin’s immunomodulatory effect on the local inflammatory milieu and immune cells responding to renal injury.

The comparison of one- and two-compartment kinetic models indicated that the two-compartment model with first-order elimination from the central compartment described rapamycin blood concentration time profiles better than the one-compartment model. The observed kinetic behavior of rapamycin is consistent with that documented in preclinical and clinical pharmacokinetic studies [44,45,46]. The results of the disposition kinetics characterization following a single IV dose of 0.1 mg/kg unformulated free rapamycin or rapamycin nanoparticles in C57BL/6 mice demonstrated that the blood concentration time profile of rapamycin follows a biexponential time course typical of two-compartment disposition. The biexponential decline in rapamycin blood concentrations after IV dosing is in part attributable to its high red blood cell partitioning [47]. Rapamycin is highly lipophilic, with a log P of 4.3 [48]. The lipophilic nature of rapamycin allows it to bind extensively to red blood cells and tissues, resulting in a plasma-to-blood partitioning ratio of 0.025~0.09 and a large volume of distribution [47,49,50]. The compartmental analysis of individual rapamycin concentration time profiles revealed that the volume of distribution and total clearance of rapamycin were significantly increased and the half-life of the fast disposition phase (*t*_1/2,*α*_) was significantly shorter in the rapamycin nanoparticle group compared with those in the unformulated free rapamycin group. The shorter *t*_1/2,*α*_ value represents a relatively rapid drug distribution from blood to tissue. The increased volume of distribution and total clearance values are likely associated with the increased drug partitioning into both non-eliminating and eliminating tissues, such as the liver and spleen, when rapamycin is delivered on PFC nanoparticles. When unformulated free rapamycin is delivered systemically, it binds to red blood cells and features a fraction unbound in blood as low as 0.1% [51,52]. However, the PFC nanoparticles retained the rapamycin on their lipid membrane and prevented the partition of rapamycin into red blood cells, which manifested a marked impact on the tissue-to-blood distribution equilibrium and the volume of distribution of rapamycin. Moreover, the increased fraction of rapamycin that was not bound to red blood cells may have resulted in an increase in the total clearance of rapamycin. In the rapamycin PFC nanoparticle group, both the volume of distribution and the total clearance of rapamycin were increased to a similar extent. Accordingly, no significant difference in the half-life of slow disposition phase (*t*_1/2,*β*_) was observed between the unformulated free rapamycin and rapamycin nanoparticle groups. While we observed a relatively short half-life during the fast disposition phase, as consistently reported in previous studies, we noticed the reported half-life reported in the slow disposition phase ranged from 3 to 79 h. Our previous study of the non-invasive pharmacokinetic characterization of rapamycin PFC nanoparticle, which utilized the fluorine (^19^F) magnetic resonance spectroscopy to register the perfluoro-15-crown-5 ether, the core of the PFC nanoparticles,, reported the half-life of the slow disposition phase as about 3 h when the administration dose is 0.07 mg/kg [38]. Other studies reported a rapamycin half-life of 5.8 h [53] or 15 h [54] with unknown dosages. It has also been reported that rapamycin’s half-life is about 79 h, when given orally at about 0.17 mg/kg in humans [55]. Since the drug half-life is dosage-dependent, the previously published results indicated that our observed slow disposition phase half-live is in the correct area. To assess the biodistribution, we examined rapamycin concentrations in selected organs 20 h after the single IV dosing of unformulated free rapamycin or rapamycin nanoparticles in C57BL/6 mice. The unformulated free rapamycin group exhibited significantly higher rapamycin concentrations in most organs, including the liver, kidney, bladder, spleen, stomach, heart, and lung, compared with the rapamycin nanoparticle group. This can be attributed to a significantly higher rapamycin blood concentration for the unformulated free rapamycin group. To determine whether rapamycin nanoparticles could alter rapamycin distribution in various organs without considering the effect of rapamycin blood concentrations on its tissue concentrations, we calculated the rapamycin tissue-to-blood concentration ratio so that any differences in the blood concentrations were accounted for in the ratio. Our data showed that the pattern of tissue-to-blood concentration ratios observed in the rapamycin nanoparticle group was similar to the pattern observed in the unformulated free rapamycin group. No significant difference in the tissue-to-blood concentration ratios was found between the two study groups in all the selected organs. These results imply that under the condition in which equivalent systemic exposures of rapamycin are achieved, rapamycin nanoparticles can provide comparable initial tissue exposure relative to unformulated free rapamycin. Therefore, the delivery of rapamycin by using PFC nanoparticles would reduce the systemic exposure of rapamycin due to the increased total clearance of drug without compromising organ delivery. Moreover, the sustained local biovailability of rapamycin delivered by PFC nanoparticles is likely enhanced by particle trapping and slow release kinetics to improve pharmacodynamics at lower effective systemic doses of the agent.

To investigate the safety of rapamycin PFC nanoparticles, we evaluated the effects on cell viability and function of vital organs when a single dose of treatment was administered. The direct exposure of endothelial cells and renal proximal tubule cells to rapamycin PFC nanoparticles did not affect their viability. Table 1 shows that liver function was not impaired according to normal total protein (normal range: 3.5–7.2 g/dL), aspartate aminotransferase (AST, normal range: 54–298 U/L), and alanine aminotransferase (ALT, normal range: 17–77 U/L). Kidney function was also not affected by rapamycin PFC nanoparticles, according to normal BUN levels (normal range: 8–33 mg/dL). Furthermore, the electrolytes on the blood test were all within the normal range: sodium was 146–151 mmol/L, potassium was 3.8–4.4 mmol/L, and chloride was 88–110 mmol/L. As for the blood cell/platelet counts, our results indicated that rapamycin PFC nanoparticles did not introduce any alterations compared to the free rapamycin treatment.

Although it is rare, cisplatin has been reported to reduce ejection fraction in patients when used as monotherapy [56]. Moreover, when combined with other cardiotoxic anti-cancer treatment, patients can develop paroxysmal supraventricular tachycardia [57]. Other studies indicate that as early as 24 h post-cisplatin treatment, testicular cancer patients receiving cisplatin developed endothelial dysfunction and vascular injury [58]. An early cross-sectional follow-up study published in 2008 suggested that testicular cancer survivors continued to experience endothelial dysfunction and vascular injury, which could put them at higher risk of cardiovascular complications [59]. A recent 30 years follow-up study on testicular cancer survivors suggested that testicular cancer survivors experienced worse diastolic function [60]. However, we [38] and others [61,62,63,64] have also documented protective effects of rapamycin on aged and injured hearts. In light of these prior observations, we used transthoracic echocardiography to confirm normal left ventricular ejection fraction and myocardial tissue strain parameters after the delivery of rapamycin-loaded nanoparticle.

Because rapamycin can feature potent immunosuppressive properties, we investigated the effects of multiple doses (once a week for four weeks) of rapamycin PFC nanoparticles on immune system function. Repeated rapamycin PFC nanoparticle administration did not significantly affect the spleen B and T cell subpopulations but did reduce the number of CD4^+^Foxp3^+^ and NK1.1^+^ cells. It is known that NF-κB signaling is critical in regulating the transition from NK1.1^−^ to NK1.1^+^ cells [65]. The inhibition of the NF-κB signaling pathway by rapamycin PFC nanoparticles may explain the lower number of NK1.1^+^ cells due to the inhibition of the transition of NK1.1^−^ cells to NK1.1^+^ cells. CD4^+^Foxp3^+^ cells are a subpopulation of CD4+ regulatory T cells (Treg). It has been reported that Foxp3 expression on Treg cells is NF-κB-dependent [66]. It is possible that rapamycin PFC nanoparticles, by inhibiting NF-κB signaling pathway activation, could affect Foxp3 expression in CD4^+^ cells. Additionally, Foxp3^+^CD4^+^ T cells play important roles in immune suppression, partially by inhibiting NF-κB signaling pathway activation [67]. Thus, a reduction in CD4^+^Foxp3^+^ cells could result in reduced systemic immune suppression through NF-κB signaling. It is possible that NF-kB depletion might result in inadequate surveillance against noxious agents and cancer [68], but further elucidation of the safety consequences of rapamycin PFC nanoparticles in reducing splenic CD4^+^Foxp3^+^ cells might be prudent. Furthermore, rapamycin PFC nanoparticle treatment did not affect T cell proliferation and their molecular responses to stimulation, nor did it elicit humoral immune responses.

## 5. Conclusions

Our results demonstrate that rapamycin PFC nanoparticles exhibited improved pharmacokinetics for the systemic delivery of rapamycin and the potential to mitigate cisplatin-induced acute kidney injury. In general, rapamycin PFC nanoparticles are safe in terms of vital organ function and normal systemic immune responses. However, additional investigation may be prudent for elucidating their effects on certain splenocyte subpopulations, Foxp3^+^CD4^+^ T cells, and NK1.1^+^ cells with repeated dosing.

## Figures and Tables

**Table 1:** Blood chemistries after free rapamycin or rapamycin PFC nanoparticle treatments.

Test Name	Free Rapamycin	Rapamycin NP
WBC (×10^3^/μL)	2.12 ± 0.38	2.12 ± 0.38
RBC (×10^6^/μL)	7.76 ± 0.10	7.79 ± 0.27
Hemoglobin (g/dL)	11.16 ± 0.22	11.14 ± 0.27
Hematocrit (%)	38.60 ± 1.04	37.94 ± 1.49
MCV (fL)	49.70 ± 0.73	48.68 ± 0.69
MCH (pg)	14.38 ± 0.30	14.32 ± 0.24
MCHC (g/dL)	28.96 ± 0.82	29.44 ± 0.83
Platelets (×10^3^/μL)	703.00 ± 57.56	794.00 ± 30.84
Blood Urea Nitrogen (mg/dL)	22.00 ± 9.84	16.80 ± 0.49
Creatinine (mg/dL)	0.48 ± 0.21	0.43 ± 0.02
Total Protein (g/dL)	7.20 ± 3.22	7.16 ± 0.15
AST (U/L)	71.6 ± 32.02	72.00 ± 13.22
ALT (U/L)	60.40 ± 27.01	70.80 ± 6.53
Sodium (Na^+^) (mmol/L)	140.25 ± 1.03	138.60 ± 0.24
Potassium (K^+^) (mmol/L)	4.35 ± 0.29	4.24 ± 0.11
Chloride (Cl^−^) (mmol/L)	107.00 ± 0.71	106.40 ± 0.40

## Data Availability

The data presented in this study are available in the article or Supplementary Materials.

## Supplementary Materials

The following supporting information can be downloaded at https://www.mdpi.com/article/10.3390/nano12030336/s1. Table S1: Systemic disposition kinetics of rapamycin in mice after single IV bolus injection of 0.1 mg/kg of unformulated rapamycin and rapamycin nanoparticle; Table S2: Comparison of rapamycin tissue concentrations obtained at 20 h post dose and the corresponding tissue-to-blood concentration ratio values between unformulated rapamycin and rapamycin nanoparticle groups.
