# Supplementary material for: Safety Profile of Rapamycin Perfluorocarbon Nanoparticles for Preventing Cisplatin-Induced Kidney Injury
Source: Nanomaterials (Basel). 2022 Jan 21;12(3):336. doi: 10.3390/nano12030336 (PMC8839776; doi:10.3390/nano12030336)
Supplement: Supplementary file 1 [file nanomaterials-12-00336-s001.zip › nanomaterials-1536391-supplementary.pdf]

Supplementary Materials

Safety Profile of Rapamycin Perfluorocarbon Nanoparticles for Preventing Cisplatin-Induced Kidney Injury

Qingyu Zhou [1,*], Justin Doherty [2], Antonina Akk [3], Luke E. Springer [3], Ping Fan [4], Ivan Spasojevic [4], Ganesh V. Halade [2], Huanghe Yang [4], Christine T.N. Pham [3,5], Samuel A. Wickline [2,6] and Hua Pan [2,*]

[1] Department of Pharmaceutical Sciences, Taneja College of Pharmacy, University of South Florida, Tampa, FL 33612, USA;
[2] USF Health Heart Institute, University of South Florida, Tampa, FL 33602, USA.; justindohert@usf.edu (J.D.); ghalade@usf.edu (G.V.H.); wickline@usf.edu (S.A.W.)
[3] Department of Medicine, Washington University School of Medicine, St. Louis, MO 63110, USA.; antoninaakk@wustl.edu (A.A.); lspringer@wustl.edu (L.E.S.); cpham@wustl.edu (C.T.N.P.)
[4] School of Medicine, Duke University, Durham, NC 27708, USA.; ping.fan@duke.edu (P.F.); ivan.spasojevic@duke.edu (I.S.); huanghe.yang@duke.edu (H.Y.)
[5] John Cochran Veterans Affairs Medical Center, St. Louis, MO 63106, USA
[6] Altamira Therapeutics Inc, Dover, DE 19901, USA
* Correspondence: qzhou1@usf.edu (Q.Z.); huapan@usf.edu (H.P.); Tel.: 1-813-974-7081 (Q.Z.); Tel.: 1-813-396-9755 (H.P.)

Table S1. Systemic disposition kinetics of rapamycin in mice after single IV bolus injection of 0.1 mg/kg of unformulated rapamycin and rapamycin nanoparticle

	**Unformulated Rapamycin (N = 7)**	**Rapamycin Nanoparticles (N = 5)**
**A (μg/L)**	352 ± 86.9	162 ± 35.0 ***
**B (μg/L)**	130 ± 41.2	37.0 ± 14.6 ***
**AUC (μg·h/L)**	2021 ± 713	423 ± 117 ***
**$t_{1/2,\alpha}$ (h)**	1.02 ± 0.33	0.34 ± 0.12 ***
**$t_{1/2,\beta}$ (h)**	7.97 ± 2.51	6.78 ± 1.51
**V_C (L/kg)**	0.237 ± 0.071	0.557 ± 0.129 ***
**V_{ss} (L/kg)**	0.448 ± 0.083	2.00 ± 0.678 ***
**V_β (L/kg)**	0.633 ± 0.137	2.57 ± 0.817 ***
**CL (L/h/kg)**	0.060 ± 0.022	0.263 ± 0.063 ***

Note: Data are presented as mean ± standard deviation (SD). ***$P < 0.001$ compared with the unformulated free rapamycin group using the independent sample *t* test for difference in means between two groups.

Table S2. Comparison of rapamycin tissue concentrations obtained at 20 hours post dose and the corresponding tissue-to-blood concentration ratio values between unformulated free rapamycin and rapamycin nanoparticle groups

	**Rapamycin Concentration (ng/mL blood or ng/g tissue)**		**Tissue-to-plasma concentration ratio**	
	**Unformulated Rapamycin (N = 4)**	**Rapamycin Nanoparticles (N = 5)**	**Unformulated Rapamycin (N = 4)**	**Rapamycin Nanoparticles (N = 5)**
**Blood**	16.2 ± 7.58	4.73 ± 1.58 *	---	---
**Liver**	14.5 ± 5.29	4.13 ± 0.83 **	1.05 ± 0.69	0.974 ± 0.387
**Kidney**	26.0 ± 6.35	10.2 ± 1.62 ***	1.73 ± 0.48	2.35 ± 0.73
**Lung**	83.0 ± 18.2	26.3 ± 3.93 ***	5.52 ± 1.29	6.52 ± 3.66
**Heart**	47.7 ± 13.3	20.9 ± 3.75 **	3.20 ± 1.14	4.79 ± 1.46
**Brain**	3.98 ± 6.02	0.149 ± 0.036	0.35 ± 0.58	0.038 ± 0.025
**Intestine**	64.4 ± 88.8	5.04 ± 1.53	5.45 ± 8.64	1.37 ± 1.14
**Stomach**	27.3 ± 11.0	8.02 ± 2.02 **	1.95 ± 1.29	2.03 ± 1.26
**Spleen**	39.8 ± 22.4	12.2 ± 3.90 *	2.81 ± 2.32	2.74 ± 0.93
**Bladder**	55.6 ± 20.6	11.7 ± 8.54 **	3.72 ± 1.56	3.56 ± 4.51

Note: Data are presented as mean ± standard deviation (SD). *P < 0.05, **P < 0.01, ***P < 0.01 compared with the unformulated rapamycin group using the independent sample *t* test for difference in means between two groups.
